# Human Bone Marrow-Derived Mesenchymal Stromal Cells Differentially Inhibit Cytokine Production by Peripheral Blood Monocytes Subpopulations and Myeloid Dendritic Cells

**DOI:** 10.1155/2015/819084

**Published:** 2015-04-28

**Authors:** Paula Laranjeira, Joana Gomes, Susana Pedreiro, Monia Pedrosa, Antonio Martinho, Brigida Antunes, Tania Ribeiro, Francisco Santos, Rosario Domingues, Manuel Abecasis, Helder Trindade, Artur Paiva

**Affiliations:** ^1^Blood and Transplantation Center of Coimbra, Portuguese Institute of the Blood and Transplantation, Quinta da Vinha Moura, São Martinho do Bispo, 3041-861 Coimbra, Portugal; ^2^QOPNA, Department of Chemistry, University of Aveiro, 3810-193 Aveiro, Portugal; ^3^Signal Transduction Laboratory, Center of Cellular Biology, SACS and Department of Biology, University of Aveiro, 3810-193 Aveiro, Portugal; ^4^Cell2B Advanced Therapeutics, SA, Biocant Park, Núcleo 04, Lote 4A, 3060-197 Cantanhede, Portugal; ^5^Serviço de Transplantação de Progenitores Hematopoiéticos (UTM), Instituto Português de Oncologia de Lisboa Francisco Gentil, Rua Professor Lima Basto, 1099-023 Lisboa, Portugal

## Abstract

The immunosuppressive properties of mesenchymal stromal/stem cells (MSC) rendered them an attractive therapeutic approach for immune disorders and an increasing body of evidence demonstrated their clinical value. However, the influence of MSC on the function of specific immune cell populations, namely, monocyte subpopulations, is not well elucidated. Here, we investigated the influence of human bone marrow MSC on the cytokine and chemokine expression by peripheral blood classical, intermediate and nonclassical monocytes, and myeloid dendritic cells (mDC), stimulated with lipopolysaccharide plus interferon (IFN)*γ*. We found that MSC effectively inhibit tumor necrosis factor- (TNF-) *α* and macrophage inflammatory protein- (MIP-) 1*β* protein expression in monocytes and mDC, without suppressing CCR7 and CD83 protein expression. Interestingly, mDC exhibited the highest degree of inhibition, for both TNF-*α* and MIP-1*β*, whereas the reduction of TNF-*α* expression was less marked for nonclassical monocytes. Similarly, MSC decreased mRNA levels of interleukin- (IL-) 1*β* and IL-6 in classical monocytes, CCL3, CCL5, CXCL9, and CXCL10 in classical and nonclassical monocytes, and IL-1*β* and CXCL10 in mDC. MSC do not impair the expression of maturation markers in monocytes and mDC under our experimental conditions; nevertheless, they hamper the proinflammatory function of monocytes and mDC, which may impede the development of inflammatory immune responses.

## 1. Introduction

Mesenchymal stromal/stem cells (MSC) correspond to undifferentiated cells capable of self-renewal and to differentiate along different cell lineages [1]. The detailed study of their immunophenotypic characteristics facilitated MSC identification, quantification, and isolation from different human adult tissues, such as bone marrow, adipose tissue, and muscle, among others [1–4]. In turn, the discovery of their immunosuppressive potential converted them into an attractive therapeutic approach for autoimmune diseases and pathological conditions where the activation of the immune system entails deleterious effects.

In the recent years, an increasing number of studies have reported the inhibitory effect of MSC over immune cells, wherein the majority of them focused on T lymphocytes [5, 6]. However, even concerning T cells, only a limited number of studies analyzed and compared the influence of MSC over distinct functional T cell subsets and demonstrated that functional T cells subsets are in fact differentially regulated by MSC [4, 6–11]. In turn, as antigen-presenting cells have a pivotal role in T cell activation, in T cell differentiation, and in directing their polarization [12], the study of MSC influence over monocytes and dendritic cells (DC) became an active field of research. Nevertheless, the number of studies performed in natural occurring DC is scarce [13, 14], as the majority of them was carried out in monocyte-derived DC differentiated* in vitro* with GM-CSF and IL-4. Furthermore, to the best of our knowledge, no study investigated and compared the influence of MSC over the recently identified peripheral blood classical, intermediate, and nonclassical monocyte subpopulations [15].

In 2010, Ziegler-Heitbrock and colleagues [15] identified three distinct subpopulations within peripheral blood monocytes, which are phenotypically and functionally characterized as follows: classical monocytes are phenotypically characterized as CD14^++^CD16^−^ [15, 16]; intermediate monocytes, identified by CD14^++^CD16^+^ phenotype, display the highest expression of class II major histocompatibility complex (MHC), compared to the remaining peripheral blood monocyte subpopulations, together with an increased ability to present antigens to T cells and to induce antigen-specific secretion of interleukin- (IL-) 12 and interferon (IFN)*γ*; besides, when challenged with zymosan or lipopolysaccharide (LPS), this cell subset presents the highest expression level of IL-10 [15, 16]; finally, nonclassical monocytes, phenotypically characterized as CD14^+^CD16^++^, are the most predisposed to differentiate into DC and induce the highest rates of T cell proliferation; however, after stimulation with LPS and IFN*γ*, they are less efficient in producing IL-1*β*, IL-6, IL-12, and tumor necrosis factor- (TNF-) *α* compared to classical monocytes and myeloid dendritic cells (mDC) [15–18]. Of note, macrophages derived from CD16^+^ monocytes possess higher phagocytic activity than those generated from classical monocytes [16].

mDC correspond to a peripheral blood subset of DC, which are likely to be in transit from the bone marrow to tissues, where they will contact with foreign antigens and undergo maturation. Accordingly, peripheral blood mDC share some characteristics with immature DC, such as antigen uptake, processing, and presentation activity, followed by T cell activation, the lack of CD83 and the production of IL-1*β*, IL-6, IL-12, and TNF-*α* after activation with LPS and IFN*γ* [12, 15, 17–21]. Of note, depending on the stimulus, mDC can acquire an anti-inflammatory expression profile, reducing IL-12 while increasing IL-10 expression, thus inducing a Th2 immune response [12, 20]. It was recently described that peripheral blood mDC can be phenotypically distinguished in two subpopulations, CD1c (BDCA-1)^+^ and CD141 (BDCA-3)^+^, with remarkable functional differences [15, 20, 21].

In the present study, we investigated influence of human bone marrow-derived MSC on peripheral blood monocyte subpopulations (classical, intermediate, and nonclassical monocytes) and mDC, stimulated with LPS and IFN*γ*, in a coculture system. With this aim, we proceeded to the quantification, by flow cytometry, of TNF-*α*, CCL4 (macrophage inflammatory protein (MIP-) 1*β*), CCR7, CD83, and HLA-DR in the abovementioned immune cell populations. mRNA expression of IL-1*β*, IL-6, CCL3 (MIP-1*α*), CCL5 (regulated on activation, normal T cell expressed, and secreted; RANTES), CXCL9 (monokine induced by gamma interferon; MIG) and CXCL-10 (interferon-gamma-inducible protein- (IP-) 10) was evaluated in previously purified classical and nonclassical monocytes, as well as IL-1*β* and CXCL10 in purified mDC. Besides, we assessed protein and mRNA expression of immune mediators and adhesion molecules in nonstimulated MSC or after LPS and/or IFN*γ* treatment.

## 2. Material and Methods

### 2.1. Collection and Isolation of Peripheral Blood Mononuclear Cells and Bone Marrow Mesenchymal Stromal Cells

Peripheral blood samples from a total of six healthy donors (1 male and 5 females; mean age of 44 ± 7 years, ranging from 22 to 51 years old), collected in heparin at the Blood and Transplantation Center of Coimbra (Portugal), and human bone marrow (BM) samples from healthy donors (age ranging from 20 to 40 years old) were included in the present study. The use of these biological samples for research purpose was approved by Serviço de Transplantação de Progenitores Hematopoiéticos (UTM) do Instituto Português de Oncologia de Lisboa Francisco Gentil (laws 97/95 and 46/2004), and all participants gave written informed consent before entering in the study.

Peripheral blood mononuclear cells (MNC) were isolated by Lymphoprep (Stemcell Technologies, Vancouver, Canada) gradient density centrifugation and then washed twice in Hank's Balanced Salt Solution (HBSS, Gibco, Life Technologies, Paisley, UK). The MNC pellet was resuspended in RPMI 1640 with GlutaMax medium (Invitrogen, Life Technologies) with antibiotic-antimycotic (Gibco), to the final concentration of 10^6^ cells/500 *μ*L.

Peripheral blood MNC were subsequently analyzed for protein and mRNA expression in the following experimental conditions: (1) 10^6^ MNC + 500 *μ*L RPMI (negative control); (2) 10^6^ MNC + 0.5 × 10^6^ MSC (negative control); (3) 10^6^ MNC + LPS + IFN*γ* (positive control); (4) 10^6^ MNC + 0.5 × 10^6^ MSC + LPS + IFN*γ*; (5) 10^6^ MNC + 0.5 × 10^6^ MSC, followed by MSC depletion and, then, stimulation with LPS + IFN*γ*. All the aforementioned cell cultures were carried out for 20 hours at 37°C, in a sterile environment with 5% CO_2_ and humidified atmosphere, plus an incubation period of 6 hours with the stimulator agents. The cell culture and stimulation protocols are detailed below, in “Immunophenotypic study of MSC and peripheral blood monocytes and mDC” section.

For the isolation of human BM-MSC, MNC were isolated from BM samples by using a Sepax S-100 system (Biosafe, Eysins, Switzerland) in accordance with the instructions of the manufacturer. Cell number and viability were determined using the Trypan Blue (Gibco) exclusion method.

BM MNC were plated at a density of 2 × 10^5^ cells/cm^2^ in Dulbecco's Modified Eagle Medium (DMEM, Gibco) supplemented with 10% qualified fetal bovine serum (FBS, Sigma, Spain). After 3-day incubation at 37°C in humidified atmosphere containing 5% CO_2_, the nonadherent cell fraction was discarded, and the adherent culture was maintained with a complete medium renewal every 3-4 days. After reaching a 70–80% confluency cells were detached using TrypLE (Life Technologies) for 7 minutes and then replated at an initial density of 3000 cells/cm^2^. For this study, MSC passages 3 and 5 were used.

MSC identity was confirmed by performing fluorescent morphological analysis, mesodermal differentiation assays (osteogenic, adipogenic, and chondrogenic), and immunophenotype characterization as described by Dominici et al. [22].

Subsequently, MSC were resuspended in RPMI 1640 with GlutaMax medium (Invitrogen) with antibiotic-antimycotic (Gibco) to the final concentration of 0.5 × 10^6^ cells/500 *μ*L. The protein and mRNA expression of MSC were studied in the following experimental conditions: (1) 0.5 × 10^6^ MSC + 500 *μ*L RPMI (nonstimulated MSC); (2) 0.5 × 10^6^ MSC + 500 *μ*L RPMI + LPS; (3) 0.5 × 10^6^ MSC + 500 *μ*L RPMI + IFN*γ*; and (4) 0.5 × 10^6^ MSC + 500 *μ*L RPMI + LPS + IFN*γ*. All the aforementioned cell cultures were carried out for 20 hours at 37°C, in a sterile environment with 5% CO_2_ and humidified atmosphere, plus an incubation period of 6 hours with the stimulator agents. The cell culture and stimulation protocols are detailed below, in “Immunophenotypic study of MSC and peripheral blood monocytes and mDC” section.

### 2.2. Immunophenotypic Study of MSC and Peripheral Blood Monocytes and mDC

*MSC Stimulation with LPS, IFNγ, and LPS + IFNγ*. For the immunophenotypic study of MSC, we plated in 8 wells of tissue culture plates (Falcon, Becton Dickinson Biosciences, BD, San Jose, USA) 0.5 × 10^6^ MSC in 1 mL of RPMI 1640 with GlutaMax medium (Invitrogen) with antibiotic-antimycotic (Gibco). MSC were cultured for 20 hours at 37°C, in a sterile environment with 5% CO_2_ and humidified atmosphere (to be in the same experimental conditions than those MSC cocultured with MNC). Then, MSC were stimulated with LPS (100 ng/mL) from* Escherichia coli* (serotype 055:B5, Sigma), IFN*γ* (100 U/mL, Promega, Madison, USA), or LPS + IFN*γ* (100 ng/mL and 100 U/mL, resp.), in duplicate (to perform the immunophenotypic study and mRNA expression quantification), for 6 hours at 37°C in humidified atmosphere containing 5% CO_2_; in the remaining 2 wells, MSC were not stimulated.

*Immunophenotypic Study of MSC*. For each experimental condition tested, cells were detached using TrypLE (Gibco); after incubating for 10 minutes at −20°C, the content of each well was transferred to a 12 × 75 mm polystyrene cytometer tube, centrifuged for 5 minutes at 540 ×g and the supernatant was discarded. MSC immunophenotype was assessed using the 7-color monoclonal antibody (mAb) combination detailed in Table 1 (tube 1). The cell pellet was incubated with the mAb for 10 minutes in the darkness and washed with phosphate buffered saline (PBS). Finally, cells were resuspended in 500 *μ*L of PBS and immediately acquired in a FACSCanto II (BD) flow cytometer.

*Coculture of Peripheral Blood MNC and MSC and In Vitro Stimulation with LPS + IFNγ*. In 6 wells of tissue culture plates (Falcon) 10^6^ MNC were plated in 1 mL of RPMI 1640 with GlutaMax medium (Invitrogen) with antibiotic-antimycotic (Gibco), and in 9 wells of tissue culture plates (Falcon) we plated 10^6^ MNC + 0.5 × 10^6^ MSC in a final volume of 1 mL, establishing a ratio of 2 : 1 (MNC : MSC). Cells were cultured for 20 hours at 37°C, in a sterile environment with 5% CO_2_ and humidified atmosphere. The content of 3 wells with cocultured MNC + MSC was subjected to MSC depletion, using the EasySep Human CD271 Selection kit (Stemcell Technologies), according to the manufacturer's instructions.

Then, LPS + IFN*γ* (100 ng/mL and 100 U/mL, resp.) were added to 3 wells with MNC, 3 wells with cocultured MNC + MSC and to the 3 wells with MNC + MSC where MSC had been depleted; and the cells in the remaining wells (3 with MNC and 3 with MNC + MSC) were not stimulated. To one of the wells in each experimental condition (MNC, MNC + MSC, MNC + LPS + IFN*γ*, MNC + MSC + LPS + IFN*γ*, MNC + MSC + Depetion + LPS + IFN*γ*), we added brefeldin A (10 *μ*g/mL) from* Penicillium brefeldianum* (Sigma), to prevent the release of* de novo* produced cytokines outside the cells. The samples were incubated at 37°C, in a sterile environment with 5% CO_2_ humidified atmosphere, for 6 hours.

The samples with brefeldin A were used for the study of TNF-*α* and MIP-1*β* expression in monocytes and mDC, by flow cytometry, while the expression of CD83, CCR7, and HLA-DR, by flow cytometry, and the mRNA expression of cytokines, in monocytes and mDC, were performed in the samples without brefeldin A. All the aforementioned protein and mRNA expression studies were performed in all the different culture conditions: MNC, MNC + MSC, MNC + LPS + IFN*γ*, MNC + MSC + LPS + IFN*γ*, MNC + MSC + Depletion + LPS + IFN*γ*.

*Immunophenotypic Study of Peripheral Blood Monocytes and mDC.* For each experimental condition tested, cells were detached using TrypLE (Gibco); after incubating for 10 minutes at −20°C, the content of each well was transferred to a 12 × 75 mm polystyrene cytometer tube, centrifuged for 5 minutes at 540 ×g, and the supernatant was discarded. Immunophenotypic analysis of peripheral blood monocytes and mDC, cultured in the presence/absence of LPS + IFN*γ* and in the presence/absence of MSC, was performed using 8-color mAb combinations, detailed in Table 1. For the study of CD83, CCR7, and HLA-DR expression (tube 2), cultured cells were incubated with the mAb for 10 minutes in the darkness, washed with PBS and, finally, resuspended in 500 *μ*L of PBS and immediately acquired in a FACSCanto II (BD) flow cytometer. To study TNF-*α* and MIP-1*β* expression (tube 3), cells were stained with the mAb for surface proteins antigens (CD16, CD45, CD33, CD14, IREM-2, and HLA-DR) and, after an incubation period of 10 minutes in the darkness, washed with PBS. For intracellular staining, Fix&Perm (Caltag, Hamburg, Germany) reagent was used, according to the manufacturer's instructions and in parallel with the mAb for TNF-*α* and MIP-1*β*. After washing twice with PBS, the cell pellet was resuspended in 500 *μ*L of PBS and immediately acquired.

*Data Acquisition and Analysis.* Data acquisition was performed in a FACSCanto II (BD) flow cytometer equipped with the FACSDiva software (v6.1.2; BD). For both MSC and MNC immunophenotypic studies, the whole sample from each tube was acquired and stored, corresponding to a number of events always above 0.1 × 10^6^ or 0.5 × 10^6^ events, respectively. For data analysis, the Infinicyt (version 1.7) software (Cytognos SL, Salamanca, Spain) was used.

**Figure 1:**
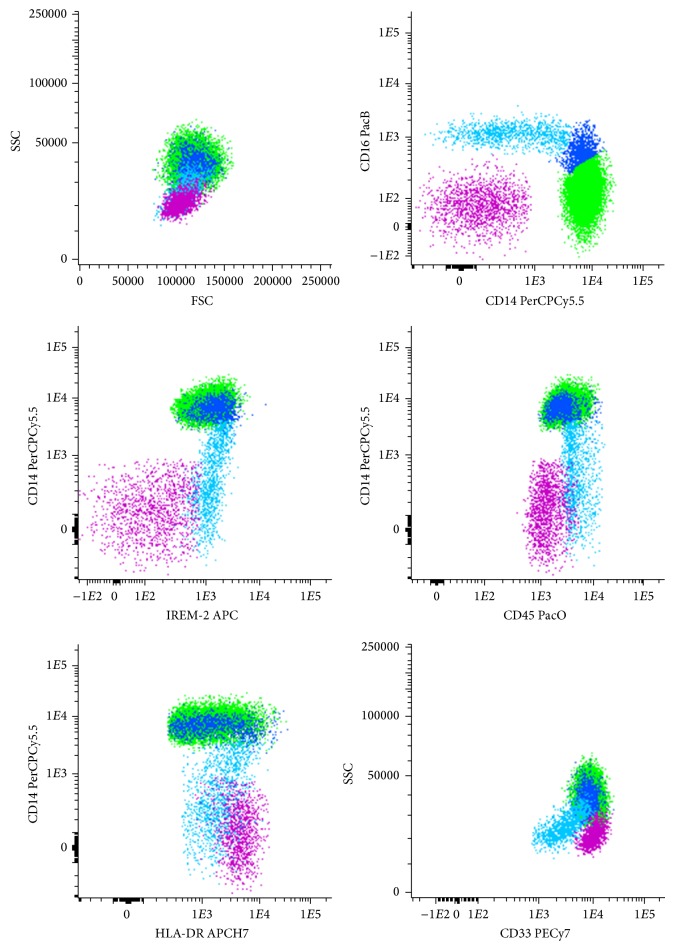
Phenotypic characteristics of peripheral blood classical, intermediate, and nonclassical monocytes and mDC. Bivariate dot plot histograms illustrating the phenotypic strategy for the identification of the different monocyte subpopulations and myeloid dendritic cells (mDC) from peripheral blood. Classical monocytes (green events) express CD14 in the absence of CD16, they also show high reactivity for CD45, CD33, IREM-2, and HLA-DR; intermediate monocytes (dark blue events) are characterized as CD14-positive displaying an increasing positivity to CD16, together with a high expression of IREM-2 and positivity for CD45, CD33, and HLA-DR; nonclassical monocytes (light blue events) are CD16-positive with a decreasing expression of CD14, they are highly positive to IREM-2 and CD45, while presenting the lowest CD33 expression among monocytes subpopulations; mDC (pink events) are phenotypically characterized as negative for CD14, CD16, and IREM-2, they present lower expression of CD45 and SSC properties and higher expression of HLA-DR and CD33, compared to monocytes.

*Immunophenotypic Identification of Classical, Intermediate, and Nonclassical Monocytes and mDC.* Attending to the markers used to analyze the expression of CCR7, CD83, HLA-DR, TNF-*α*, and MIP-1*β* in classical, intermediate and nonclassical monocytes and mDC, we used the following gate strategy to identify these four cell populations (Figure 1): classical monocytes express high levels of CD14 in the absence of CD16, together with high expression of CD33 and HLA-DR and being also positive for IREM-2 (CD300e); intermediate monocytes express high levels of CD14 as well, but display an increasing expression of CD16, associated to a slight decrease of CD33 expression, compared to classical monocytes; in turn, nonclassical monocytes show CD16 positivity with a decreasing expression of CD14, they present the highest expression of CD45 along with the lowest expression of CD33 among the three monocyte subpopulations, and HLA-DR and IREM-2 expression is between that of classical and intermediate monocytes; mDC have lower side-scatter light dispersion properties and CD45 expression than monocytes, they present high expression of CD33 and HLA-DR in the absence of CD14, CD16, and IREM-2.

### 2.3. Cell Purification by Fluorescence-Activated Cell Sorting

Monocytes and mDC cell populations from the cell cultures were purified by fluorescence-activated cell sorting, using FACSAria II flow cytometer (BD), according to their typical phenotype. Thus, the 6-color mAb combination used (Table 1, tube 4) allowed the identification of classical monocytes (HLA-DR^+^CD33^+^IREM-2^+^CD14^++^CD16^−^), nonclassical monocytes (HLA-DR^+^CD33^+^IREM-2^+^CD14^+^CD16^+^), and mDC (HLA-DR^++^CD33^++^IREM-2^−^CD14^−^CD16^−^). The purified cell populations were subsequently used for the quantification of mRNA expression.

### 2.4. Analysis of mRNA Expression in MSC and Peripheral Blood Monocyte and mDC

The content of each well of cultured MSC under the different experimental condition tested or the purified mDC and monocyte subpopulations was transferred to a 1.5 mL eppendorf tube, centrifuged for 5 minutes at 300 ×g and the pellet resuspended in 350 *μ*L of RLT Lysis Buffer (Qiagen, Hilden, Germany). Total RNA was extracted with the RNeasy Micro kit (Qiagen), according to the supplier's instructions. Then, total RNA was eluted in a 20 *μ*L volume of RNase-free water RNA was reverse transcribed with Tetra cDNA Synthesis (Bioline, London, UK), according to the manufacturer's instructions. Relative quantification of gene expression by real-time PCR was performed in the LightCycler 480 II (Roche Diagnostics, Rotkreuz, Switzerland). Real-time PCR reactions were carried out using 1x QuantiTect SYBR Green PCR Master Mix (Qiagen), 1x QuantiTect Primer Assay (for MSC: TNF3: QT01079561; IL-8: QT00000322; IL-6: QT00013461; ICOSL: QT00023660; IDO: QT00000504; TGF-*β*1: QT00025718; IL-1*β*: QT00021385; for purified classical and non-classical monocytes and mDC: IL-1*β*: QT00021385; IL-6: QT00083720; CCL3: QT01008063; CCL5: QT00090083; CXCL9: QT00013461; CXCL10: QT01003065) (Qiagen), in a final volume of 10 *μ*L. The reactions were performed using the following thermal profile: 1 cycle of 10 min at 95°C, 50 cycles of 10 sec at 95°C, 20 sec at 55°C, and 30 sec at 72°C, 1 cycle of 5 sec at 95°C, 1 min at 65°C and continuo at 97°C, and 1 cycle of 10 sec at 21°C. All samples were run in duplicate. Real-time PCR results were analyzed with the LightCycler software (Roche Diagnostics). GeNorm software (PrimerDesign Ltd., Southampton, England) was used to select the reference genes to normalize data. The reference genes used for gene expression analysis were cytochrome c1 (CYC1) and splicing factor 3a subunit 1 (SF3A1) for MSC; glyceraldehyde-3-phosphate dehydrogenase (GAPDH) and topoisomerase DNA I (TOP1) for classical monocytes; and GAPDH and beta-2 microglobulin (B2M) for nonclassical monocytes and mDC. The normalized expression levels of the genes of interest were calculated by using the delta-Ct method.

### 2.5. IDO Detection by Western Blot

Indoleamine-2,3-dioxygenase (IDO) protein levels expression were determined by western blot in MSC without stimulation cultured in the presence or absence of MNC and in MSC stimulated with LPS, IFN*γ* or LPS + IFN*γ*, in the presence or absence of MNC.

Cell lysates were prepared using RIPA buffer (Sigma) supplemented with complete protease inhibitor cocktail (Roche). After centrifugation at 10,000 ×g for 10 min at 4°C, supernatants were collected. Seeblue Plus2 Prestained Protein Standard (Invitrogen) and Precision Plus Protein Standard (BioRad, Warszawa, Poland) were used as standard. SDS-polyacrylamide gel electrophoresis was performed for separation of the proteins, and western blot was subsequently performed. For protein specific detection, the membranes were incubated with the primary mAb anti-IDO (clone 10.1, Merck Millipore, Darmstadt, Germany) diluted 1 : 5000.

### 2.6. IL-6 Production Analysis

IL-6 production was determined by enzyme-linked immunosorbent assay (ELISA), using a commercially available ELISA kit (RayBiotech, Norcross, USA), in the supernatant of MSC cultured in the presence/absence of MNC, with or without stimulation by LPS, IFN*γ* or LPS + IFN*γ*. The cultured cells were pelleted and the media was recovered to be tested by ELISA, performed according to the manufacturer's instructions.

### 2.7. Statistical Analyses

To determine the statistical significance of the differences observed between different culture conditions, nonparametric Friedman's paired-sample test was performed, using Statistical Package for Social Sciences (IBM SPSS, version 17.0, Armonk, NY, USA). Data were expressed as mean percentage ± standard deviation. Statistically significant differences were considered when *P* value was lower than 0.05.

## 3. Results

In order to better understand how MSC regulate the immune function of the recently described monocyte subpopulations and mDC, we evaluated the expression of proteins involved in cell migration, activation/maturation, antigen presentation, and the production of proinflammatory cytokines, in LPS plus IFN*γ* stimulated monocytes and mDC in the presence or absence of MSC.

### 3.1. MSC Inhibit TNF-*α* and MIP-1*β* Protein Expression in Monocytes and mDC

**Figure 2:**
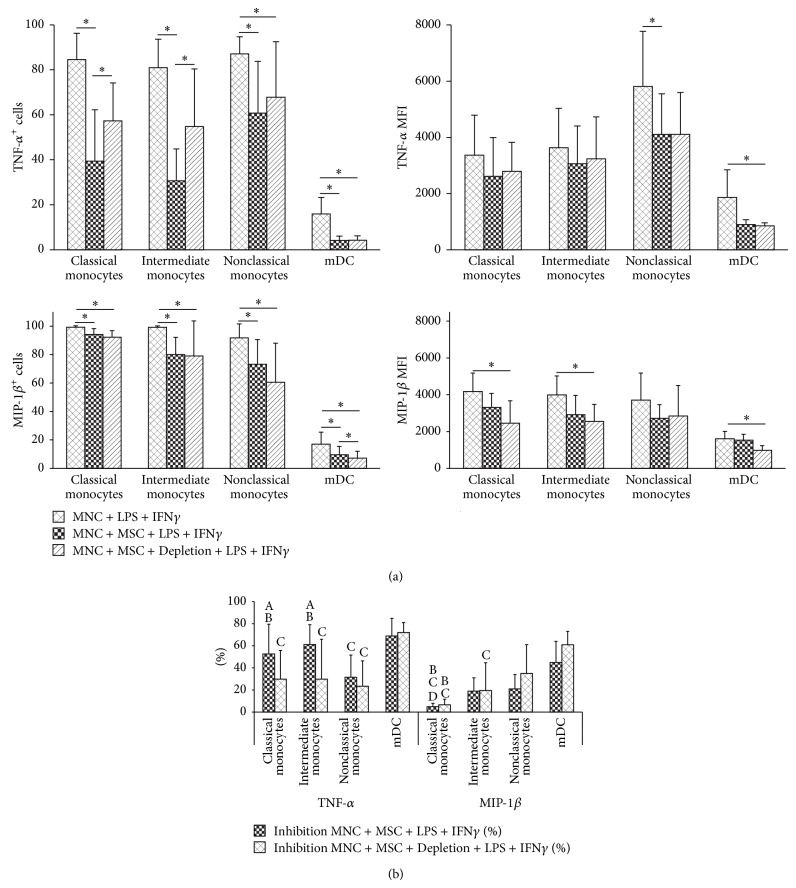
MSC inhibition of TNF-*α* and MIP-1*β* protein expression in monocytes and mDC. (a) Percentage (mean ± standard deviation) of cells producing TNF-*α* and MIP-1*β* and amount of protein expressed per cell, measured as mean fluorescence intensity (MFI, mean ± standard deviation), for classical, intermediate, and nonclassical monocytes and mDC, under the following culture conditions: upon MNC stimulation with LPS + IFN*γ* (MNC + LPS + IFN*γ*), MNC cocultured with MSC and stimulated with LPS + IFN*γ* in the presence of MSC (MNC + MSC + LPS + IFN*γ*), MNC cocultured with MSC and stimulated with LPS + IFN*γ* immediately after depletion of MSC from the culture system (MNC + MSC + Depletion + LPS + IFN*γ*). Statistically significant differences were considered when *P* < 0.05 (∗) for Friedman's paired-sample test. (b) Percentage (mean ± standard deviation) of inhibition by MSC (present in the culture system during LPS + IFN*γ* MNC activation or depleted prior to activation) on the percentage of monocytes and mDC producing TNF-*α* and MIP-1*β*. Statistically significant differences were considered when *P* < 0.05 for Friedman's paired-sample test: ^A^versus the same cell population in MNC + MSC + Depletion + LPS + IFN*γ* condition; ^B^versus nonclassical monocytes in the same culture conditions; ^C^versus mDC in the same culture conditions; ^D^versus intermediate monocytes in the same culture conditions. MSC, mesenchymal stromal cells; TNF-*α*, tumor necrosis factor-*α*; MIP-1*β*, macrophage inflammatory protein-1*β*; mDC, myeloid dendritic cells; LPS, lipopolysaccharide; IFN*γ*, interferon *γ*; MNC, mononuclear cells.

Considering TNF-*α* and MIP-1*β* expression, our results showed that MSC decreased both the percentage of cells producing TNF-*α* and MIP-1*β* (*P* < 0.05, for all cell populations) as well as the amount of protein produced per cell (measured as mean fluorescence intensity, MFI, *P* < 0.05 for TNF-*α*-producing nonclassical monocytes and mDC, and for MIP-1*β*-producing classical and intermediate monocytes and mDC), wherein MSC depletion prior to LPS + IFN*γ* stimulation resulted in a less effective reduction of the percentage of TNF-*α* producing monocytes (Figure 2(a)). It is worth mentioning that MSC inhibition capability is different for distinct proinflammatory proteins and for the different cell populations addressed in this study: MSC are less effective in regulating MIP-1*β* than TNF-*α*, and the expression of TNF-*α* in nonclassical monocytes and of MIP-1*β* in classical monocytes is inhibited to a lesser extent than that of the remaining cell populations (Figure 2(b)). Furthermore, mDC exhibited the highest percentage of inhibition among all cell populations under study (Figure 2(b)). We found a similar behavior for MSC + MNC cocultures stimulated solely with LPS (data not shown). Transwell assays demonstrated that the regulatory effect of MSC over TNF-*α* and MIP-1*β* was partially due to soluble factors (data not shown).

### 3.2. MSC Influence CCR7 and CD83 Protein Expression in Monocytes and mDC

**Figure 3:**
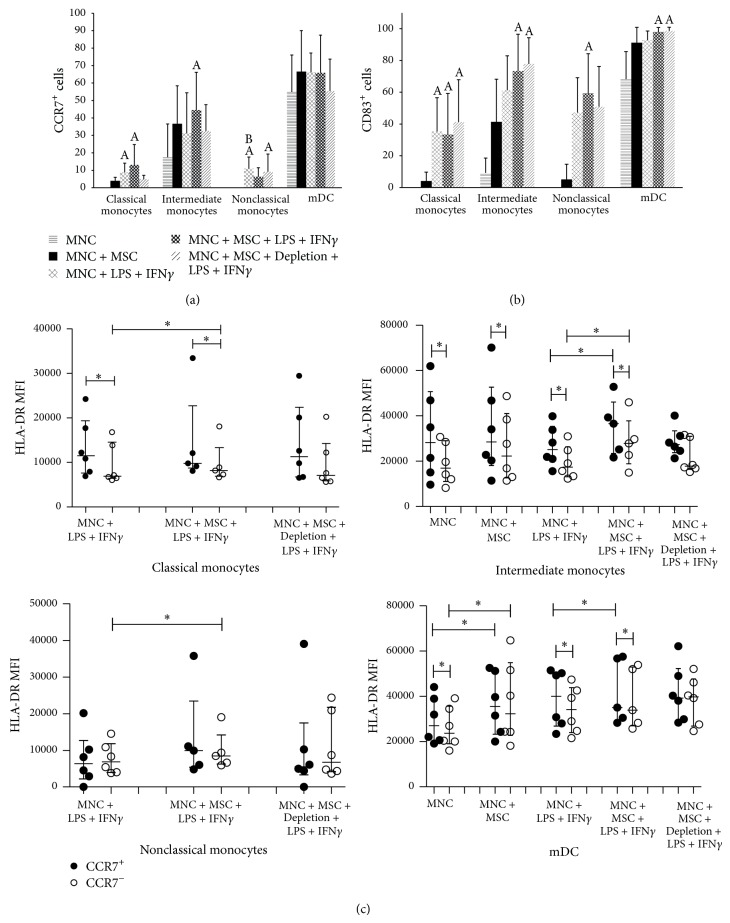
Influence of MSC on CCR7, CD83, and HLA-DR protein expression in monocytes and mDC. Percentage (mean ± standard deviation) of CCR7^+^ (a) and CD83^+^ (b) cells among classical, intermediate, and nonclassical monocytes and mDC, under the following culture conditions: nonstimulated MNC (MNC), nonstimulated MNC cocultured with MSC (MNC + MSC), MNC stimulated with LPS + IFN*γ* (MNC + LPS + IFN*γ*), MNC cocultured with MSC and stimulated with LPS + IFN*γ* in the presence of MSC (MNC + MSC + LPS + IFN*γ*), MNC cocultured with MSC and stimulated with LPS + IFN*γ* immediately after depletion of MSC from the culture system (MNC + MSC + Depletion + LPS + IFN*γ*). Statistically significant differences were considered when *P* < 0.05 for Friedman's paired-sample test: ^A^versus MNC; ^B^versus MNC + MSC. (c) Expression of HLA-DR (measured as mean fluorescence intensity) among CCR7^+^ and CCR7^−^ monocyte subpopulations and mDC. Statistically significant differences were considered when *P* < 0.05 (∗) for Friedman's paired-sample test. MSC, mesenchymal stromal cells; mDC, myeloid dendritic cells; MNC, mononuclear cells; LPS, lipopolysaccharide; IFN*γ*, interferon *γ*.

In peripheral blood, classical, intermediate and nonclassical monocytes and mDC from healthy individuals do not express CCR7 nor CD83; however, 24-hour culture of MNC in RPMI induced CCR7 and CD83 expression in a small percentage of intermediate monocytes and in an important percentage of mDC, further increased when cocultured with MSC (Figures 3(a) and 3(b)). For classical and nonclassical monocyte subpopulations, CCR7 and CD83 were only expressed after LPS + IFN*γ* stimulus. Of note, the simultaneous presence of MSC and LPS + IFN*γ* generated an even higher percentage of CCR7^+^ cells for classical and intermediate monocytes and also of CD83^+^ cells for latter cell population. Interestingly, if MSC were depleted prior to LPS + IFN*γ* stimulation, the percentage of CCR7^+^ cells was similar to that observed in MNC + LPS + IFN*γ* condition for both monocyte subpopulations (Figure 3(a)). We found no important alterations in the amount of CCR7 expressed per cell (MFI) among the different culture conditions, for all cell populations considered; however, an increased CD83 MFI was observed in intermediate and nonclassical monocytes and mDC in MNC + MSC + LPS + IFN*γ* culture condition (data not shown). To further confirm that CCR7^+^ monocytes and mDC corresponded to cells undergoing maturation, we evaluated the amount of HLA-DR expressed per cell (MFI), because mature monocytes and mDC express higher levels of HLA-DR. Accordingly, we found that HLA-DR expression (MFI) was higher for CCR7^+^ versus CCR7^−^ cells, for classical and intermediate monocytes subpopulations and mDC, reaching statistical significance (Figure 3(c)).

### 3.3. MSC Differentially Regulate Cytokine and Chemokine mRNA Expression in mDC, Classical, and Nonclassical Monocytes

**Figure 4:**
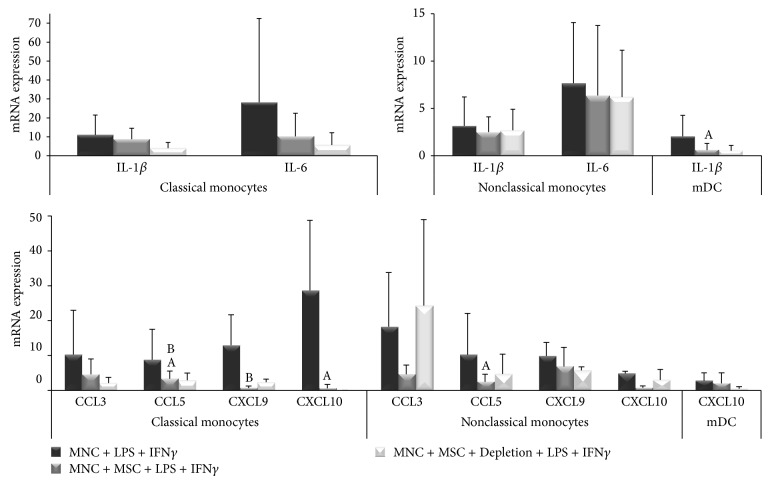
MSC effect on cytokines and chemokines mRNA expression in monocytes and mDC. Semiquantitative analysis of IL-1*β*, IL-6, CCL3, CCL5, CXCL9, and CXCL10 mRNA expression in classical and nonclassical monocytes and mDC, under the following culture conditions: MNC stimulated with LPS + IFN*γ* (MNC + LPS + IFN*γ*), MNC cocultured with MSC and stimulated with LPS + IFN*γ* in the presence of MSC (MNC + MSC + LPS + IFN*γ*), MNC cocultured with MSC and stimulated with LPS + IFN*γ* immediately after depletion of MSC from the culture system (MNC + MSC + Depletion + LPS + IFN*γ*). Statistically significant differences were considered when *P* < 0.05 for Friedman's paired-sample test: ^A^versus MNC + LPS + IFN*γ*; ^B^versus MNC + MSC + Depletion + LPS + IFN*γ*. MSC, mesenchymal stromal cells; mDC, myeloid dendritic cells; IL, interleukin; MNC, mononuclear cells; LPS, lipopolysaccharide; IFN*γ*, interferon *γ*.

The regulation of IL-1*β*, IL-6, CCL3, CCL5, CXCL9, and CXCL10 mRNA expression by monocytes and mDC was also evaluated. MSC showed a high ability to reduce mRNA expression of all the cytokines and chemokines under study in classical monocytes (*P* < 0.05 for CCL5 and CXCL10) (Figure 4); remarkably, the mRNA expression of IL-1*β*, IL-6, CCL3, and CCL5 was even lower for MSC + MNC coculture in which MSC were depleted before MNC stimulation with LPS + IFN*γ* (*P* < 0.05 for CCL5), except for CXCL9 (*P* < 0.05). However, for nonclassical monocytes, only the mRNA expression of chemokines was significantly downregulated (*P* < 0.05 for CCL5, Figure 4); whereas, in mDC, IL-1*β* mRNA expression was downregulated (*P* < 0.05) and CXCL10 remained unchanged under the influence of MSC (Figure 4). Remarkably, MSC depletion prior to LPS + IFN*γ* stimulation abrogated CCL3 and diminished CCL5 and CXCL10 mRNA regulation for non-classical monocytes, while decreasing CXCL10 mRNA expression in mDC (Figure 4).

### 3.4. LPS and IFN*γ* Modulate mRNA and Protein Expression in MSC

**Figure 5:**
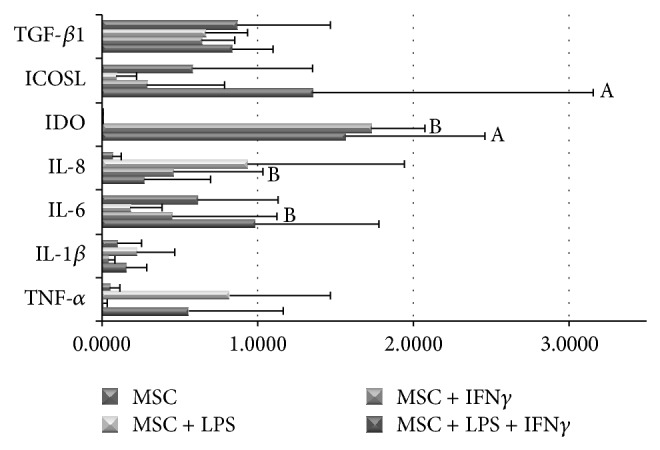
Modulation of mRNA expression of pro- and anti-inflammatory proteins in MSC by LPS, IFN*γ* and LPS + IFN*γ*. Semiquantitative analysis of TGF-*β*, ICOSL, IDO, IL-8, IL-6, IL-1*β*, and TNF-*α* mRNA expression in MSC after stimulation with LPS, IFN*γ*, or LPS + IFN*γ*. Statistically significant differences were considered when *P* < 0.05 for Friedman's paired-sample test: ^A^versus MNC; ^B^versus MNC + LPS. MSC, mesenchymal stromal cells; LPS, lipopolysaccharide; IFN*γ*, interferon *γ*; TGF-*β*, transforming growth factor *β*; ICOSL, inducible costimulatory ligand; IDO, indoleamine-2,3-dioxygenase; IL, interleukin; TNF-*α*, tumor necrosis factor-*α*; MNC, mononuclear cells.

To understand how the MNC stimulators used in this study affect MSC activity, we evaluated the expression of molecules with an important role in the immune function, at mRNA and protein level, in nonstimulated MSC and after LPS and/or IFN*γ* stimulation (Figure 5).

MSC showed constitutive expression of CD13, CD44, CD73, CD90, CD106, CD184, and IL-6 proteins and IL-6, inducible costimulatory ligand (ICOSL) and transforming growth factor *β* (TGF-*β*1) mRNA transcripts (Figure 5). The variation of the expression of the proteins assessed by flow cytometry is expressed here as the ratio of the (MFI of MSC + stimulator)/(MFI of nonstimulated MSC). The protein expression of CD73 and CD184 increased after LPS (ratio of 1.47 ± 1.10 and 1.28 ± 0.55, resp.), IFN*γ* (ratio of 1.24 ± 0.74 and 1.23 ± 0.35, resp.), and LPS + IFN*γ* (ratio of 1.34 and 1.49, resp.); CD90 increased after treatment with LPS or LPS + IFN*γ* (ratio of 1.64 ± 1.16 and 1.49 ± 0.70, resp.); whereas only the stimulation of MSC with LPS + IFN*γ* increased the protein expression of CD44 and CD106 (ratio of 1.22 ± 0.24 and 1.30 ± 0.47, resp.); finally, CD13 expression showed no alterations after treatment with the stimulator agents. None of the described results reached statistical significance.

At mRNA level, we verified that LPS + IFN*γ* stimulation induced upregulation of TNF-*α*, IL-1*β*, IL-6, IL-8, IDO, and ICOSL. Besides, MSC stimulation with LPS increased the mRNA expression of TNF*α* and IL-1*β*, while decreasing ICOSL; an upregulation of IL-8 mRNA was observed after stimulation with either LPS or IFN*γ*; and IDO mRNA levels increased in MSC stimulated with IFN*γ* (Figure 5). However, the analysis of the supernatant from MSC cultures by ELISA, showed that IL-6 was constitutively expressed by MSC and neither LPS nor IFN*γ* altered their expression at protein level. Additionally, IDO was not detected by western blot in MSC lysates (data not shown) and no alterations were found in TGF-*β*1 mRNA expression after MSC stimulation with LPS and/or IFN*γ* (Figure 5). None of the stimulators altered MSC morphology nor differentiation capability; and though MSC stimulated with LPS showed a higher level of expansion, it occurred after the first passage and after culture day 10, thus not affecting our results (data not shown).

## 4. Discussion

The aim of the present work was to gain a deeper insight on the immunosuppressive effect of human bone marrow-derived MSC on the recently described peripheral blood monocyte subpopulations (classical CD14^++^CD16^−^, intermediate CD14^++^CD16^+^, and nonclassical CD14^+^CD16^++^ monocytes) [15, 16] and mDC. To the best of our knowledge, this is the first study reporting the effect of MSC on these specific cell populations. To accomplish this goal, the expression of TNF-*α*, CCL4 (MIP-1*β*), CCR7, and CD83 was evaluated by flow cytometry and the mRNA expression of IL-1*β*, IL-6, CCL3 (MIP-1*α*), CCL5 (RANTES), CXCL9 (MIG), and CXCL10 (IP-10) was quantified in FACS-sorted and purified classical and nonclassical monocytes, and IL-1*β* and CXCL10 mRNA levels were evaluated in mDC.

Finally, we evaluated protein and mRNA expression in nonstimulated MSC and after LPS and/or IFN*γ* treatment, in order to understand how their regulatory function could be affected. As previously described, we found a slight increase in the mRNA of the proinflammatory cytokines TNF-*α* and IL-6, along with a significant increase in the chemotactic IL-8 [23–29] and the immunosuppressor IDO, ICOSL, and CD73 [26, 30, 31], upon stimulation with LPS + IFN*γ*. Of note, an increased expression of other immunoregulatory molecules, such as fibroblast growth factor 2 (FGF2), hepatocyte growth factor (HGF), cyclooxygenase 2 (COX2), prostaglandin E2 (PGE2), upon TLR4 stimulation had also been described in the literature [26, 28, 32, 33]. Moreover, LPS + IFN*γ* promoted an increased protein expression of adhesion molecules in MSC: CD106 (VCAM-1), CD184 (CXCR4), CD54 (ICAM-1), CD90 (Thy-1), and CD44. These adhesion molecules are involved in cell-to-cell and cell-extracellular matrix interaction and their upregulation had been reported to improve both MSC interaction with immune cells, namely, T cells, and MSC migration [34–37]. Taken together these data suggest that, under an inflammatory microenvironment, MSC increase the expression of adhesion molecules that allow their close interaction with immune cells, contributing to increase the efficiency of paracrine mediators with immunosuppressive function produced by MSC.

Our data showed that MSC efficiently inhibit TNF-*α* and MIP-1*β* production, in all the three monocytes subpopulations and mDC, by reducing both the percentage of producing cells and the amount of cytokine produced per cell, albeit our results suggested that MSC exert a more pronounced inhibitory effect over TNF-*α* expression than over MIP-1*β*. The partial inhibition observed in transwell assays suggests that both soluble factors and cell contact mechanisms are involved in this process. Interestingly, MSC exerted a higher inhibition over TNF-*α* production by intermediate monocytes and mDC (compared to classical and nonclassical monocytes), which correspond to the two cell populations more skilled for antigen presentation to T cells [16]. Furthermore, MSC impaired the mRNA expression of all the cytokines and chemokines under study in classical monocyte, as well as of the chemokines in nonclassical monocytes and of IL-1*β* in mDC. Taken together, our data support that MSC inhibit the proinflammatory function of monocytes and mDC both by reducing the expression of proinflammatory cytokines (TNF-*α*, IL-1*β*, and IL-6) and also by downregulating chemotactic factors that attract T cells and monocytes (CCL3, CCL4, CCL5, CXCL9, and CXCL10) and whose expression is induced by LPS plus IFN*γ*.

In fact, LPS binding to TLR4 expressed on monocytes and mDC plasmatic membrane results in the downstream activation of MAPK (ERK1/2, JNK, and p38), NF-kB, and AP-1 [38], followed by the induction of the transcription of TNF-*α*, IL-1*β*, IL-6, MIP-1*β* (CCL4), CCL3, CCL5, CXCL9, and CXCL10 [39–46]. IFN*γ* signaling can also contribute to IL-1*β*, CXCL9, and CXCL10 mRNA expression in human monocytes, macrophages and DC [41, 47–49]. Despite the overlapping signaling molecules participating on TNF-*α* and MIP-1*β* gene expression induction [39], MSC showed more efficiency in inhibiting TNF-*α*, suggesting differences between these two signaling pathways.

Taking into account previous studies which investigated the mechanisms underlying the inhibition of cytokine and chemokine expression by MSC, we can point out the constitutively expressed IDO, Jagged-1, adenosine, and/or PGE2 as possible candidates to the impairment of TNF-*α* production observed in the cells under study [13, 28, 50–55]; whereas PGE2 has also been described to reduce MIP-1*β* expression in monocyte-derived DC [56, 57]. As, for the evaluation of protein expression by flow cytometry, we added brefeldin A to the cell culture together with LPS + IFN*γ* (which stopped the proteins synthesized* de novo* in the Golgi apparatus and prevented them to reach the extracellular medium), the inhibition of TNF-*α* and MIP-1*β* production can only be attributed to proteins constitutively expressed by MSC or induced after their contact with peripheral blood MNC.

Conversely, the assays made to evaluate mRNA expression were conducted in the absence of brefeldin A, so, for the final inhibitory effect accounted proteins constitutively expressed by MSC or induced by the contact with MNC, stimulator agents, or cytokines produced by MNC in response to LPS plus IFN*γ* activation. In fact, stimulation of MSC with LPS and/or IFN*γ* induces the expression of several immunosuppressor factors, such as HGF, nitric oxide (NO) and insulin-like growth factor (IGF) and increase Jagged-1, PGE2, and IDO expression [25, 26, 31–33]. In line with this, previous studies demonstrated that IL-6 expression might be inhibited by Jagged-1 and HGF in macrophages [55, 58], or by IDO in DC [53], though IDO presents the opposite effect in human cancer cells [59]. In turn, PGE2 has the ability to impair the expression of CCL3, CCL5, and CXCL10 in human DC, macrophages [56, 60, 61], microglial cells and astrocyes [62] and of CXCL9 and CXCL10 in human breast cancer cells [48]; HGF and NO were demonstrated to inhibit CCL5 expression in human renal tubular epithelial cells and in mouse keratinocytes, respectively [63–65], while CXCL10 expression in melanoma cells is downregulated by NO [66]. The decreased expression of genes induced by TLR4 signaling pathway was shown to result from ERK1/2 inhibition by Jagged-1, and NF-kB inhibition by PGE2, HGF, IDO, and adenosine [51, 53–55, 58, 63, 67].

In the present study, neither CCR7 nor CD83 protein expression was inhibited by the presence of MSC in the cell culture; furthermore, CCR7^+^ cells displayed a higher HLA-DR MFI than their CCR7^−^ counterparts; altogether suggesting that MSC do not inhibit monocyte and mDC activation/maturation under our experimental conditions. Regarding the important role of CCR7 in monocyte and DC migration, it was demonstrated that solely the upregulation of CCR7 expression does not necessarily corresponds to increased migration ability; however, it has been reported that PGE2 can upregulate the expression of CCR7, CD83, and HLA-DR in DC and of CCR7 in monocytes and, simultaneously, increase the cells' migratory response to the lymph node-derived CCR7 ligands, CCL19 and CCL21 [50, 57, 68–72]. IFN*γ* share the ability to increase CCR7 expression in monocyte-derived DC and, if administrated with TLR4 agonist, to potentiate their CCR7-driven migration; in turn, NO and IGF-I can induce CD83 expression in DC [49, 73, 74].

According to our data, MSC do not impede the expression of CD83 and CCR7 in LPS + IFN*γ*-activated monocyte and mDC, but impair their proinflammatory function by decreasing the expression of TNF-*α*, IL-1*β*, and IL-6. In the same line, the reduction of chemokine expression induced by MSC may inhibit classical and nonclassical monocytes' ability to recruit T cells and monocytes, consequently hampering the interaction of these antigen-presenting cells with T cells.

It was recently reported that macrophages and monocytes can assume immunoregulatory functions, induced by IL-6 and/or PGE2, and characterized by decreased TNF-*α* expression and increased IL-10 production [30, 75–78], wherein MSC can actively participate [79, 80]. Attending to the particular experimental conditions of the present study, as reported here and by others, MSC constitutively express IL-6 which is further upregulated upon stimulation with LPS [23, 25–28, 33] and this cytokine has the ability to downregulate TNF-*α* expression in monocytes [81, 82].

Several studies reported MSC-derived inhibition of CD83, CD80, CD86, and HLA-DR upregulation in DC differentiation assays [14, 82–87] and impairment of their migratory ability, both* in vitro* and* in vivo*, toward CCL21 [88, 89]. In opposition, Berk et al. reported that human umbilical cord blood MSC did not inhibit monocyte differentiation into immature DC and supported not only DC maturation but also the migration towards CCL21 [90].

It is worth to mention that we detected ICOSL mRNA in MSC, as recently described by Yi et al. [91], which was upregulated upon LPS + IFN*γ* stimulation. This finding opens new possibilities for the immunosuppressive mechanisms of MSC, because ICOSL induces IL-10-producing regulatory T cells [12].

Here, we did not use a protocol to differentiate monocytes into DC, instead we analyzed the naturally occurring peripheral blood monocytes subpopulations and mDC (which corresponds to a more physiological condition) to assess the influence of MSC. Our results suggest that, upon MNC stimulation, MSC do not impede monocyte or mDC to express the activation/maturation markers CD83, CCR7 and HLA-DR. Conversely, MSC impair monocyte and mDC proinflammatory function, by decreasing the production of TNF-*α*, IL-1*β*, and IL-6, and reducing the monocytes' ability to recruit T cells and other monocytes, by diminishing MIP-1*β*, CCL3, CCL5, CXCL9, and CXCL10 expression.

As MSC are highly sensitive to the microenvironment, small differences in the experimental protocol may yield different results. In fact, concentration and time of exposure to LPS may conditioned MSC influence on immune cells [25, 77, 92]; also, the state of activation/maturation of DC will determine different responses to MSC regulatory factors [50]; all these variables difficult the comparison of the results obtained in different studies.

## 5. Conclusions

Altogether, our results showed that bone marrow-derived MSC inhibit TNF-*α* and MIP-1*β* protein expression in activated classical, intermediate, and nonclassical monocytes, as well as in mDC. Remarkably, the inhibition observed was more pronounced for TNF-*α* expression than for MIP-1*β* and, regarding TNF-*α*, nonclassical monocytes were more resistant to MSC-induced suppression. Of note, mDC exhibited the highest degree of regulation for both TNF-*α* and MIP-1*β*. Similarly, MSC downregulate mRNA expression of proinflammatory cytokines and chemokines by monocytes and mDC. Conversely, the induction of activation/maturation markers (CD83, CCR7, and HLA-DR) by LPS plus IFN*γ* activation was not impaired by MSC. Thus, despite of not suppressing the activation/maturation of monocytes and mDC, MSC impair their proinflammatory function by reducing the expression of pro-inflammatory cytokines and chemotactic factors for monocytes and T cells, which may ultimately hamper the development of an inflammatory immune response.

## Supplementary Material

Material and methods for the transwell assays and for the analysis of the growth kinetics, morphology and differentiation of MSC after stimulation with LPS, IFNγ and LPS plus IFNγ.

## Figures and Tables

**Table 1:** Panel of mAb reagents (with clones and commercial source) used for the immunophenotypic characterization of classical, intermediate, and nonclassical monocytes, mDC, and MSC.

Fluorochrome								
Tube	PacB	PacO	FITC	PE	PerCPCy5.5	PECy7	APC	APCH7
	CD44	CD45	CD106	CD73	CD184	CD13	CD90	
1	IM7	HI30	51-10C9	AD2	12G5	Immu103.44	5E10	
	Biolegend	Invitrogen	BD Pharmingen	BD Pharmingen	BD	Beckman Coulter	BD Pharmingen	

	CD16	CD45	CD83	CCR7	CD14	CD33	IREM-2	HLA-DR
2	3G8	HI30	HB15a	3D12	M5E2	D3HL60.251	UP-H2	L243
	BD Pharmingen	Invitrogen	Beckman Coulter	BD Pharmingen	BD Pharmingen	Beckman Coulter	Immunostep SL	BD

	CD16	CD45	TNF-*α*	MIP-1*β*	CD14	CD33	IREM-2	HLA-DR
3	3G8	HI30	MP6-XT22	D21-1351	M5E2	D3HL60.251	UP-H2	L243
	BD Pharmingen	Invitrogen	BD Pharmingen	BD Pharmingen	BD Pharmingen	Beckman Coulter	Immunostep SL	BD

	CD16			CD123	CD14	CD33	IREM-2	HLA-DR
4	3G8			SSDCL Y107D2	M5E2	D3HL60.251	UP-H2	L243
	BD Pharmingen			Beckman Coulter	BD Pharmingen	Beckman Coulter	Immunostep SL	BD

mAb, monoclonal antibody; mDC, myeloid dendritic cells; MSC, mesenchymal stromal cells; PacB, pacific blue; PacO, pacific orange; FITC, fluorescein isothiocyanate; PE, phycoerythrin; PerCPCy5.5, peridinin chlorophyll protein-cyanine 5.5; PECy7, phycoerythrin-cyanine 7; APC, allophycocyanin; APCH7, allophycocyanin-hilite 7. Commercial sources: Biolegend (San Diego, CA, USA); Invitrogen, Life Technologies (Carlsbad, CA, USA); BD Pharmingen (San Diego, CA, USA); BD (Becton Dickinson Biosciences, San Jose, CA, USA), Beckman Coulter (Miami, FL, USA); Immunostep S.L (Salamanca, Spain).
