# Supplementary material for: Human Bone Marrow-Derived Mesenchymal Stromal Cells Differentially Inhibit Cytokine Production by Peripheral Blood Monocytes Subpopulations and Myeloid Dendritic Cells
Source: Stem Cells Int. 2015 Apr 28;2015:819084. doi: 10.1155/2015/819084 (PMC4427776; doi:10.1155/2015/819084)
Supplement: Supplementary file 1 — Material and methods for the transwell assays and for the analysis of the growth kinetics, morphology and differentiation of MSC after stimulation with LPS, IFNγ and LPS plus IFNγ. [file 819084.f1.pdf]

Supplementary methods

Analysis of the growth kinetics, morphology and differentiation of MSC after stimulation with LPS, IFNγ and LPS+IFNγ

MSCs were stimulated using LPS (100 ng/ml) from Escherichia coli (serotype 055:B5-Sigma), IFNγ (100 U/ml) and LPS+IFNγ (100 ng/ml and 100 U/ml, respectively), for 6 hours at 37ºC in humidified atmosphere containing 5% CO2.

For the analysis of the growth kinetics, after incubation with the activating agents, cells were washed twice using in DMEM supplemented with 10% FBS and seeded at 6000 cells/cm2. After 3 days in culture, cells were passed and re-seeded in a new culture flask. Cultures were passed every 7 days. The number of population doublings (PDs) was calculated according to PDs=log[(number of harvested cells)/(number of plated cells)]/log(2).

The morphological analysis was performed after the first passage post stimuli. Cells were seeded into a 24-well plate at a density of 3000 cells/cm2 for each tested condition. Cells were incubated overnight to allow adherence to the plate and washed with PBS (Gibco), fixed with 4% PFA (Sigma) solution for 30 minutes. Cell membranes were permeabilized with a saponin/PBS solution (50 µg/ml) for 45 minutes followed by incubation with Phalloidin-TRITC solution (1 µl/ml) during 60 minutes. Cells were then labeled with a solution of 1.5 µl/ml of DAPI for 5 minutes. Finally, the cells were observed under an inverted fluorescence microscope (LEICA DMI 3000B, Wetzlar, Germany) and at least 3 images per well were captured with a 100x magnification. The images were subsequently treated using the software ImageJ 1.46 Image Processing and Analysis.

MSC multilineage differentiation assays were performed in MSC stimulated with LPS+IFNγ, as described in previous work [Santos 2011], using StemPro Osteogenesis/Adipogenesis/ Chondrogenesis Differentiation Kits (Life Technologies).

Transwell assays

Peripheral blood MNC (106 cells) were placed in the upper chamber of the transwell system with 500 µl of RPMI 1640 with GlutaMax medium (Invitrogen) with antibiotic-antimycotic (Gibco), or MSC (0.5x106 cells), establishing a ratio of 2:1 (MNC:MSC), in the lower chamber. After an incubation period of 20 hours, at 37 °C, in a sterile environment with 5% CO2 humidified atmosphere, the cells in the culture were

stimulated with LPS (100 ng/ml), IFNγ (100 U/ml), or LPS+IFNγ (100 ng/ml and 100 U/ml, respectively), added to the upper chamber, as follows: 1) MNC cultivated in the absence of MSC (20h), stimulation with LPS+IFNγ (6h); 2) MNC+MSC co-culture (20h); 3) MNC+MSC co-culture (20h), MNC were removed from the transwell system and stimulated with LPS+IFNγ (6h); 4) MNC+MSC co-culture (20h), LPS stimulation (6h); 5) MNC+MSC co-culture (20h), LPS+IFNγ stimulation (6h); 6) MNC+MSC co-culture with IFNγ stimulation (20h); 7) MNC+MSC co-culture with IFNγ stimulation (20h), MNC were removed from the transwell system and stimulated with LPS+IFNγ (6h). The incubation with the stimulator agents was made in the previously described conditions. The expression of TNF-α and MIP-1β in monocytes and mDC under these culture conditions was assessed by flow cytometry.

References:

1. dos Santos F, Andrade PZ, Boura JS, Abecasis MM, da Silva CL, Cabral JM (2010) Ex vivo expansion of human mesenchymal stem cells: a more effective cell proliferation kinetics and metabolism under hypoxia. J Cell Physiol 223(1): 27-35.
